# Surgery outcomes of lamellar macular eyes with or without lamellar hole-associated epiretinal proliferation: a meta-analysis

**DOI:** 10.1186/s12886-020-01617-4

**Published:** 2020-08-25

**Authors:** Hanyue Xu, Ling Qin, Yifan Zhang, Yinan Xiao, Ming Zhang

**Affiliations:** 1 West China School of Medicine, West China Hospital, Sichuan University, Chengdu, 610041 People’s Republic of China; 2 Department of Ophthalmology, West China Hospital, Sichuan University, Chengdu No. 37, Guoxue Alley, Chengdu, 610041 People’s Republic of China

**Keywords:** Lamellar macular hole, Lamellar hole-associated epiretinal proliferation, Surgery, Best corrected visual acuity, Ellipsoid zone

## Abstract

**Background:**

Given the two different kinds of epiretinal membranes, this study aimed to compare both the structural and functional outcomes of lamellar macular holes with and without lamellar hole-associated epiretinal proliferation (LHEP) after surgery.

**Method:**

Publications up to July 2020 that compared the surgical outcomes of lamellar macular hole with and without LHEP were included. Forest plots were created by using a weighted summary of proportion meta-analysis. Fixed or random effects models were used on the basis of I2 heterogeneity estimates. Meanwhile, to evaluate the stability of the meta-analysis, a sensitivity analysis was carried out.

**Results:**

Eight pertinent publications that contained a total of 176 eyes without LHEP and 173 eyes with LHEP were included. They were all retrospective studies and had a follow-up of at least 6 months. In all studies, the preoperative best corrected visual acuity showed no significant differences between the two groups, and the visual acuity improved in both groups after surgery. The pooled result for the improved best corrected visual acuity was 0.18 (95% confidence interval (CI), 0.10 to 0.26; *P* < 0.01) between the with and without LHEP groups. The restored ellipsoid zone odds ratio was 0.80 (95% CI, 0.26 to 2.44; *P* = 0.69) for the group with LHEP compared to the group without LHEP.

**Conclusion:**

Patients without LHEP had better postoperative visual acuity than patients with LHEP. No significant difference in restored ellipsoid zone was found between the two groups.

## Background

Lamellar macular holes (LMHs), first described in biomicroscopic and angiographic findings by Gass in a case report, is a partial-thickness loss of foveal tissue [1]. The formation of LMHs is attributable to cystoid macular oedema, contraction of the perifoveal epiretinal membrane and vitreous traction [2]. Later, Witkin et al. redefined the LMH diagnosis to include (1) an irregular foveal contour; (2) a break in the inner fovea; (3) an intraretinal split; and (4) intact foveal photoreceptors [2].

The development of ultrahigh-resolution optical coherence tomography (UHR-OCT) has contributed to revealing more microscopic structures of LMHs. UHR-OCT has allowed visualization of the trapped vitreous or posterior hyaloid, termed epiretinal membranes (ERMs), in most LMH cases. Two types of ERM, tractional ERM (T ERM) and lamellar hole-associated epiretinal proliferation (LHEP), are known as thick ERMs and dense ERMs, respectively. Based on the OCT results, T ERM is described as a dense reflective line above the retina, while LHEP is a homogenous medium with much lower reflectivity. Moreover, T ERM has better contractile properties than LHEP [3]. In general, these two kinds of ERM can exist in LMH patients simultaneously or separately, and T ERM is more common than LHEP.

Whether to perform surgery on LMH patients remains controversial. Pars plana vitrectomy with internal limiting membrane (ILM) peeling, the most common surgery among LMH patients, is reportedly useful, particularly for those with significantly low visual acuity [4–6]. However, some patients did not gain better best-corrected visual acuity (BCVA) and even developed full-thickness macular holes (FTMHs) after surgery. Moreover, a previous study reported that some patients could maintain a functionally and morphologically steady state with just observation since the natural progress of the LMH was stable [7].

To determine the differences in prognosis among patients with or without LHEP, some previous studies focused on the surgical outcomes of patients with and without LHEP, but their results were inconsistent. Based on those studies, this meta-analysis aimed to compare both the structural and functional outcomes of patients with and without LHEP after surgery.

## Methods

A comprehensive search for studies that compared surgical outcomes of LMH patients with and without LHEP was carried out in the PubMed, Medline, Embase and Clinical trials databases. The search strategy was “lamellar macular hole” or “LMH” or “epiretinal membrane” or “ERM” or “lamellar hole-associated epiretinal proliferation” or “LHEP” and “surgery” or “operation” or “vitrectomy”. All references of the included articles were also screened to guarantee no omission of literature.

### Study selection

For study selection, the inclusion criteria were 1) LMH patients with T ERM or LHEP or both; 2) basic and clinical information of the patients provided; 3) evaluating surgery conducted on the patients; 4) both preoperative and postoperative BCVA reported; and 5) a median follow-up of at least 6 months. The exclusion criteria were as follows: 1) inadequate information on the included patients; 2) only patients who did not undergo surgery were observed; and 3) patients with other ophthalmologic diseases that affect the progress of LMH were included.

### Data extraction and validity assessment

All information from the included studies was separately extracted by two authors with a standardized protocol, including basic characteristics, such as authors, year, cohort size and country; detailed study information, including study design, follow-up period and surgery method; and patient information, including age, sex, and preoperative and postoperative eye-related data. The Newcastle-Ottawa Scale (NOS) was used by two authors to score the quality of all included studies separately, and disagreements were resolved by another author with more experience.

### Quantitative data synthesis

The summary odds ratios (ORs) and 95% confidence intervals (95% Cis) for LMH patients with and without LHEP were calculated, and the weighted mean difference (WMD) and 95% CI was calculated for BCVA. A *P* value < 0.05 was considered to be statistically significant. Both fixed effects and random effects models were used to pool the studies. When the *I*^2^ index, which measures the extent of the heterogeneity, was less than 50%, the conclusions were drawn from the results of the fixed effects model; otherwise, a random effects model was used. To identify potential publication bias, funnel plots were used. Sensitivity analysis was carried out to evaluate the stability of the meta-analysis by omitting one study at a time. All statistical analyses were conducted using Review Manager (version 5.2; Cochrane Collaboration, Oxford, UK; http://ims.cochrane.org/revman) and STATA software (version 11.0; Stata Corp LP, College Station, TX).

## Results

### Characteristics of the available studies

**Fig. 1:**
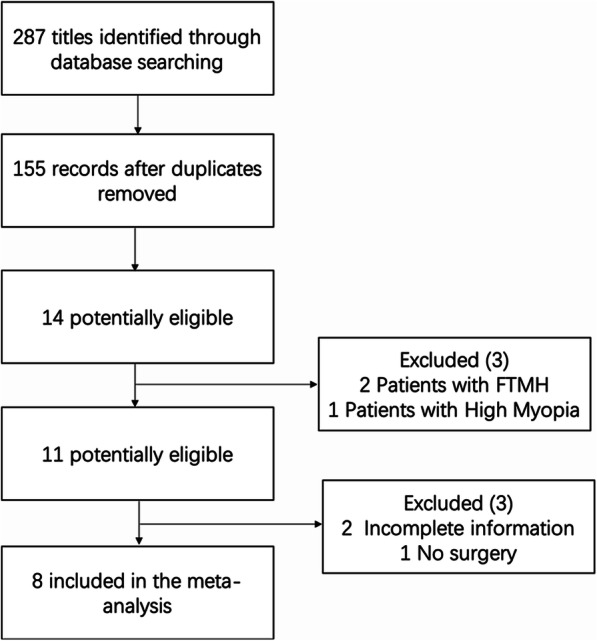
Flow diagram of the screening of the literature

A total of 287 records were obtained using the search method described above (Fig. 1). After removing duplications and articles written in other languages, 155 articles remained. By reading the titles and abstracts, 14 papers on LHEP reporting surgical outcomes were identified. Then, the full texts of all articles were read, and 6 of them were discarded because they included patients with FTMH [8, 9], recruited LMH patients with high myopia [10], had incomplete information [11, 12] or were an observational study without surgery [3]. Finally, eight studies with 176 eyes without LHEP and 173 eyes with LHEP were included [13–20]. All studies included were retrospective studies, and their detailed information is listed in Tables 1 and 2.

**Table 1:** Demographic characteristics of the included studies

Author	Parolini et al.	Lai et al.	Ko et al.	Choi et al.	Ho et al.	Takahashi et al.	Figueroa et al.	Morescalchi et al.
**Year**	2011	2015	2016	2017	2019	2019	2019	2020
**Country**	Germany	China	Korea	USA	China	Japan		Italy
**Study design**	retrospective	retrospective	retrospective	retrospective	retrospective	retrospective	retrospective	prospective
**Study period**	2008–2010	2009–2013	2011–2014	2009–2015	2013–2016	2010–2016	2010–2017	2015–2017
**Surgery**	Vitrectomy ILM/ERM peeling	Vitrectomy ILM/ERM peeling	Vitrectomy ILM/ERM peeling	Vitrectomy ILM/ERM peeling	Vitrectomy ILM/ERM peeling or LHEP embedding	Vitrectomy ILM peeling LHEP embedding	Vitrectomy ILM/ERM peeling	Vitrectomy ILM/ERM peeling
**Follow-up**	6 months	> 12 months	> 6 months	> 6 months	> 19 months	> 12 months	> 6 months	6 months
**Without LHEP**								
**Number**	6	24	58	11	–	–	77	–
**Age (Y)**	67.7 ± 12.9	59.8 ± 8.9	64.4 ± 9.5	68.6 ± 8.8	–	–	67 ± 8.9	–
**Gender (M/F)**	1/5	9/15	9/16	–			–	
**FT (**μm**)**	–	146.9 ± 51.2	154.0 ± 24.7	166.7 ± 62.0	–	–	279.1 ± 108	–
**DLMHI (**μm**)**	–	–	450.4 ± 201.8	308.2 ± 121.2	–	–	–	–
**AL (mm)**	–	–	23.66 ± 1.59	–	–	–	–	–
**With LHEP**								
**Number**	13	19	15	11	31	34	26	24
**Age (Y)**	73.9 ± 12.9	60.2 ± 11.2	67.4 ± 9.5	69.9 ± 13.6	67.7 ± 8.7	69.6 ± 10.1	67 ± 8.9	72.1 ± 8.3
**Gender (M/F)**	8/5	9/10	4/11	–	6/25	12/22	–	15/9
**FT (μm)**	–	98.4 ± 35.0	116.7 ± 38.1	96.3 ± 33.2	–	–	224 ± 66	–
**DLMHI (**μm**)**	–	–	613.5 ± 197.3	334.1 ± 139.8	–	–	–	–
**AL (mm)**	–	–	24.12 ± 1.84	–	–	24 (< 26), 10 (26≦)	–	–

*Abbreviations*: *AL* Axial Length, *DLMHI* Diameter of Lamellar Macular Hole at the ILM level (pre-operation), *ERM* Epiretinal Membrane, *FT* Foveal Thickness (pre-operation), *ILM* Internal Limited Membrane, *LHEP* Lamellar Hole-Associated Epiretinal Proliferation, *M/F* male/female

**Table 2:** Ophthalmic information of involved studies

Author	Parolini et al.	Lai et al.	Ko et al.	Choi et al.	Ho et al.	Takahashi et al.	Figueroa et al.	Morescalchi et al.
**BCVA (pre-operation)**								
Without LHEP	0.40 ± 0.20	0.72 ± 0.32	0.30 ± 0.26	0.48 ± 0.18	–	–	0.38 ± 0.19	–
With LHEP	0.40 ± 0.20	0.79 ± 0.37	0.38 ± 0.38	0.50 ± 0.25	0.37 ± 0.27	0.31 ± 0.25	0.56 ± 0.19	0.44 ± 0.16
**BCVA (post-operation)**								
Without LHEP	0.20 ± 0.30	0.43 ± 0.44	0.10 ± 0.10	0.16 ± 0.16	–	–	0.18 ± 0.17	–
With LHEP	0.20 ± 0.20	0.45 ± 0.39	0.33 ± 0.40	0.40 ± 0.29	0.17 ± 0.21	0.10 ± 0.25	0.39 ± 0.28	0.17 ± 0.13
**EZD (pre-operation)**								
Without LHEP	–	9 (24)	0 (42)	3 (11)	–	–	11 (77)	–
With LHEP	–	13 (19)	2 (10)	10 (11)	10 (33)	15 (34)	13 (26)	–
**EZD (post-operation)**								
Without LHEP	–	8 (24)	2 (42)	2 (11)	–	–	6 (77)	–
With LHEP	–	7 (19)	2 (10)	8 (11)	4 (33)	8 (34)	12 (26)	–
**CRT (pre-operation) (μm)**								
Without LHEP	–	–	–	166.7 ± 62.0	–	–	–	–
With LHEP	–	–	–	96.3 ± 33.2	–	123.2 ± 42.6	–	146 ± 34
**CRT (post-operation) (μm)**								
Without LHEP	–	–	–	230.6 ± 103.3	–	–	–	–
With LHEP	–	–	–	205.6 ± 112.9	–	191.2 ± 45.3	–	272 ± 24

*Abbreviations*: *BCVA* Best corrected visual acuity, *CRT* Central Retinal Thickness, *EZD* Ellipsoid zone destruction, *LHEP* Lamellar hole-associated epiretinal proliferation

### Outcomes of the meta-analysis

**Fig. 2:**
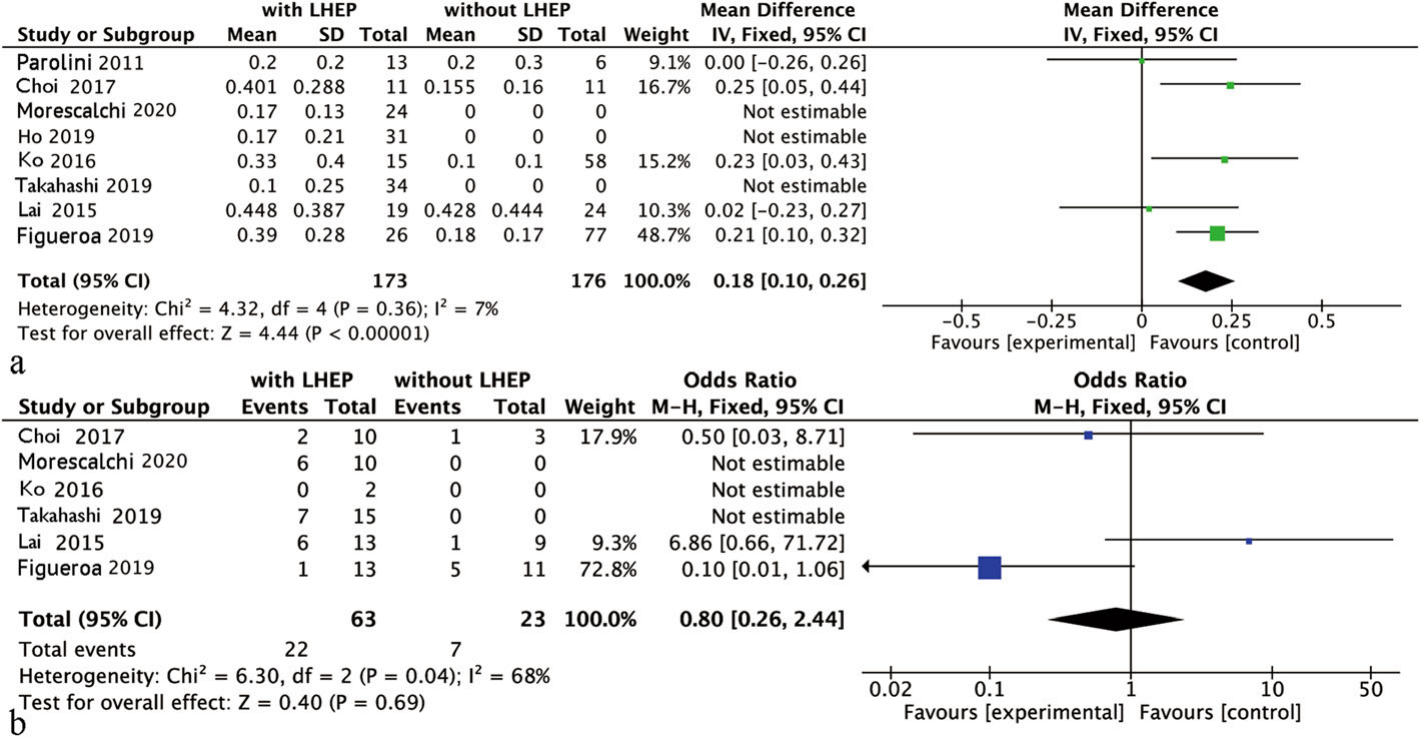
**a** Meta-analysis of the best corrected visual acuity in patients comparing eyes with and without lamellar hole-associated epiretinal proliferation (LHEP), **b** Meta-analysis of the ellipsoid zone restoration rate in patients comparing eyes with and without LHEP

No significant heterogeneity was observed in the model of BCVA (*I*^2^ = 7%, *P* = 0.36), while heterogeneity existed in the model of restored ellipsoid zone (REZ) (*I*^2^ = 68%, *P* = 0.04). Therefore, fixed-model analysis was used in the meta-analysis of BCVA, and a random-model was used for REZ. The WMD of the improved logarithm of the BCVA minimum angle of resolution between the with and without LHEP groups was 0.18 (95% CI, 0.10 to 0.26); the difference was statistically significant (*P* < 0.001). Only three studies reported the status of the ellipsoid zone both before and after surgery. The pooled data revealed an OR of 0.80 (95% CI, 0.26 to 2.44) for REZ, and the difference was not statistically significant at the 95% CI level (*P* = 0.69) (Fig. 2).

### Publication bias and validity assessment

**Fig. 3:**
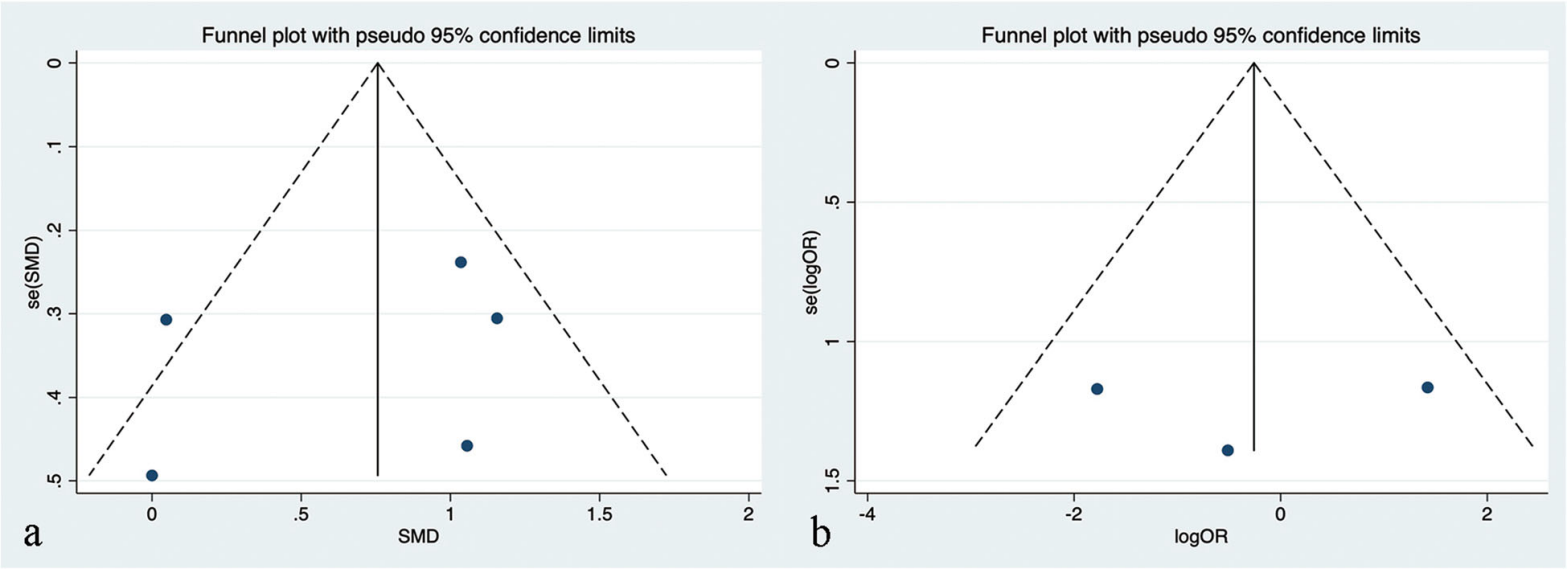
**a** The funnel plot of studies included in the analysis of BCVA, **b** the funnel plot of studies included in the analysis of ellipsoid zone restoration rate

**Fig. 4:**
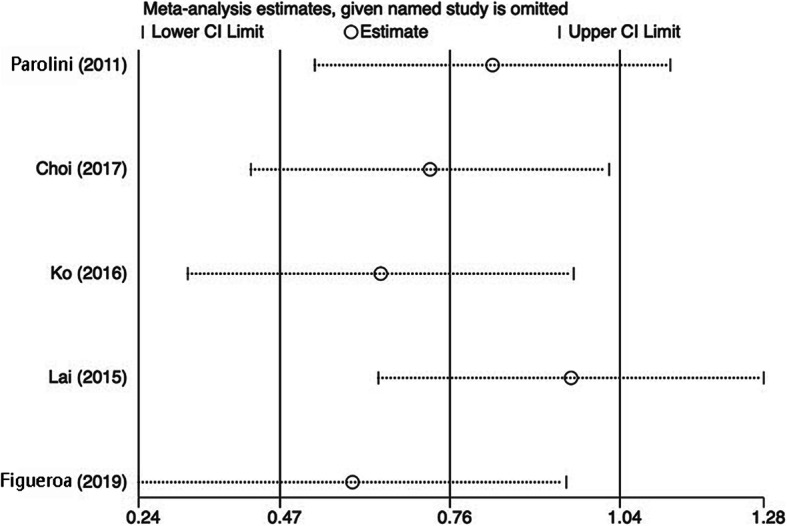
The sensitivity analysis on the pooled results of the best corrected visual acuity (BCVA)

To assess potential publication bias in the meta-analysis, funnel plots were visually inspected, and no funnel plot asymmetry was visualized (Fig. 3). By Egger’s test, no publication bias existed in the studies that reported BCVA (*P* = 0.543). Sensitivity analysis showed that the significant findings for BCVA did not change by removing any one of the studies, indicating the stability of this meta-analysis (Fig. 4). According to the NOS results (Supplementary 1), all included studies were high quality with scores equal to or greater than 7.

## Discussion

Given the different clinical characteristics of LMH patients with ERMs, surgical efficacy remains controversial. To determine whether surgery is appropriate for all LMH patients, we conducted this meta-analysis. The results suggested that although BCVA improved in all patients after surgery, BCVA in LHEP patients was lower than that in T ERM patients, and the REZ rate was not significantly different between the two groups.

The existence of ERM has been detected for several decades, while its pathogenesis and category remain unclear. T ERM is tractional, and the retinal surface under it is usually plicate. LMH with T ERM always has a short diameter and shallow cleft and is limited to the inner part of the retina; it has been described as resembling a “high hat” by Govetto et al. In contrast, LHEP is an atypical ERM without traction, having a larger and deeper LMH under it [21]. In a previous study, glial cells and hyalocytes were found in both membranes, while α-SAM was only found in T ERMs, explaining the tractional ability of T ERMs [22].

The origin of the LHEP is not clear, but there are two main theories. In one theory, as LHEP has abundant clusters of fibrous long-spacing collagen, fibroblasts and hyalocytes, the posterior detachment of the vitreous body might induce both anterior and tangential traction and therefore plays a role in the formation of ERM [16, 22]. However, in another theory, cystic spaces in LHEP formed by leakage of fluid from retinal vessels suggested that LHEP might originate from the middle retinal layers since they share the same characteristics [3]. Proof that the yellow colour of LHEP is xanthophyll mainly produced by muller cells further certifies the relationship between LHEP and the middle retinal layers [23].

Based on the results of the present study, the postoperative BCVA recovery in LHEP patients was less than that in patients without LHEP. In addition, previous studies also indicated that LMH or FTMH eyes with LHEP had worse visual outcomes after surgery [8, 13, 14, 24], probably attributable to the more severe destruction of the retina in eyes with LHEP than in those without LHEP. However, some studies have reported similar surgery outcomes between eyes with and without LHEP [10, 12, 15, 16]. As there have been no significant differences in preoperative BCVA (< 20/40), surgery method (ILM and ERM peeling) or time of follow-up (> 6 months) among these two kinds of studies, more studies with longer follow-up are required to determine the relationship between poor visual outcomes and LHEP. Although many studies have reported a positive association between the REZ and postoperative BVCA [25, 26], no significant differences in REZ existed between the with and without LHEP groups in the present study. Previous studies reported that although defection of the ellipsoid zone before surgery was associated with worse postoperative BCVA, its restoration showed no direct association with functional recovery [5, 27]. Additionally, several influential factors, including the physical conditions of the patients, differing preoperative BCVA, and varying follow-up time, should also be taken into consideration. In a previous study that had a higher REZ rate in LHEP patients, the preoperative BCVA of patients was lower than 20/40 [13], while in the other two studies, the preoperative BCVA had a wider range [14, 15].

Patients from six of the eight included studies underwent standard pars plana vitrectomy and conventional ERM and ILM peeling [13–17, 19]. However, the other two studies utilized a new surgical method [18, 20]. Comparing these two surgical methods, the difference between them lies in the disposition of ERM and ILM. Instead of peeling the membrane, the new method double inverted the ERM and flapped the ILM [28, 29]. This kind of surgery can preserve the LHEP and promote LHM recovery. A previous study indicated that the development of LHEP might be a part of the recovery progress of LMH [15]. This new surgical method and its positive outcomes raise further doubts regarding the necessity of surgery for LHM patients with ERM. Thus, determining whether surgery is necessary for patients with LHEP to obtain functional and morphological restoration requires more study.

However, our study has some limitations. First, the limited number of studies involved inevitably leads to bias, and no subgroup analysis was performed based on the different surgical methods. Thus, more studies are required to obtain more convincing results. Second, only 5 studies reported the number of cataract patients, and the number of patients with combined phacoemulsification and intraocular lens implantation may influence the BCVA outcome. Third, different choices of the gas for the final tamponade, such as air, sulfur hexafluoride, or perfluoropropane gas, may have had a distinct influence on the surgical results.

## Conclusion

This study pooled the postoperative outcomes of LMH patients with and without LHEP and found that the postoperative BCVA of patients without LHEP was better than that of patients with LHEP, and REZ showed no significant difference between the two groups.

## Supplementary information

**Additional file 1: Supplementary 1.**. Newcastle-Ottawa quality assessment T scale.

## Data Availability

The datasets used and/or analysed during the current study are available from the corresponding author on reasonable request.

## Abbreviations

BCVA: Best corrected visual acuity
CI: Confidence interval
ERM: Epiretinal membrane
DLMH: Diameter of lamellar macular hole
FTMH: Full thickness macular hole
ILM: Internal limiting membrane
LHEP: Lamellar hole-associated epiretinal proliferation
LMH: Lamellar macular hole
NOS: Newcastle-Ottawa Scale
OR: Odds ratio
REZ: Restored ellipsoid zone
SFT: Shortest foveal thickness
T ERM: Tractional epiretinal membrane
UHR-OCT: Ultrahigh-resolution optical coherence tomography
WMD: Weighted mean difference
